# Increased utilization of fructose has a positive effect on the development of breast cancer

**DOI:** 10.7717/peerj.3804

**Published:** 2017-09-27

**Authors:** Xiajing Fan, Hongru Liu, Miao Liu, Yuanyuan Wang, Li Qiu, Yanfen Cui

**Affiliations:** Laboratory of Cancer Cell Biology, Key Laboratory of Breast Cancer Prevention and Therapy, National Clinical Research Center for Cancer, Tianjin Medical University Cancer Institute and Hospital, Tianjin, China

**Keywords:** Fructose, Breast cancer, GLUT5, KHK, Metastasis

## Abstract

Rapid proliferation and Warburg effect make cancer cells consume plenty of glucose, which induces a low glucose micro-environment within the tumor. Up to date, how cancer cells keep proliferating in the condition of glucose insufficiency still remains to be explored. Recent studies have revealed a close correlation between excessive fructose consumption and breast cancer genesis and progression, but there is no convincing evidence showing that fructose could directly promote breast cancer development. Herein, we found that fructose, not amino acids, could functionally replace glucose to support proliferation of breast cancer cells. Fructose endowed breast cancer cells with the colony formation ability and migratory capacity as effective as glucose. Interestingly, although fructose was readily used by breast cancer cells, it failed to restore proliferation of non-tumor cells in the absence of glucose. These results suggest that fructose could be relatively selectively employed by breast cancer cells. Indeed, we observed that a main transporter of fructose, GLUT5, was highly expressed in breast cancer cells and tumor tissues but not in their normal counterparts. Furthermore, we demonstrated that the fructose diet promoted metastasis of 4T1 cells in the mouse models. Taken together, our data show that fructose can be used by breast cancer cells specifically in glucose-deficiency, and suggest that the high-fructose diet could accelerate the progress of breast cancer *in vivo*.

## Introduction

Breast cancer is most common in women both in the developed and the developing world (Torre et al., 2015). The American Cancer Society’s estimates showed that about 246,660 new cases would be diagnosed in the United States women in 2016, and about one in eight (12%) women would develop invasive breast cancer during their lifetime (Harbeck, 2013). Several risk factors for breast cancer have been well documented, and diet is one of the most important contributors, especially in the developed countries (Alegre et al., 2013). Thus, good eating habit could eventually have a significant impact in reducing the breast cancer incidence and progression.

Cancer cells have characteristics of active proliferation and vigorous growth, so they often process metabolic abnormalities and metabolic reprogramming to adapt to their rapid proliferation (Agnihotri & Zadeh, 2016; Beloribi-Djefaflia, Vasseur & Guillaumond, 2016). Cancer cells are prone to perform active glycolysis and generate large amounts of lactic acid even in oxygen sufficient conditions, which is the Warburg effect (Liberti & Locasale, 2016; Schwartz, Supuran & Alfarouk, 2017; Xu et al., 2015). The rapid proliferation and Warburg effect make cancer cells consume plenty of glucose, which leads to a low glucose micro-environment around cancer cells (Xu et al., 2015). To survive in micro-environment of glucose insufficiency, cancer cells could utilize other nutrients to substitute for glucose to promote their growth (Scharping & Delgoffe, 2016).

As the second largest sugar ingested in the human body, fructose is an important source of fuel in the diet especially in western diet, and fructose constitutes more than 40% of sweetener consumption in western countries, in which high-fructose corn syrup consumption increased by more than 1,000% between 1970 and 1990 (Bray, Nielsen & Popkin, 2004). In addition, fructose has the highest sweetness among all natural sugars, and its sweetness is about 1.8 times that of sucrose (Das, 2015). It is important to note that, fructose is actually more easily metabolized than glucose, because it bypasses the rate-limiting enzyme of the glycolytic pathway, and its metabolism is not controlled by insulin (Samuel, 2011).

Recent epidemiological studies have revealed a correlation between excessive fructose consumption and tumor genesis and progression (Charrez, Qiao & Hebbard, 2015; Liu & Heaney, 2011; Liu et al., 2010; Port, Ruth & Istfan, 2012). High fructose intake was associated with an increased risk of pancreatic cancer, and increased the degree of malignancy of pancreatic cancer (Hsieh et al., 2017; Li et al., 2016). Acute myeloid leukemia (AML) cells could use transporter GLUT5 to enhance fructose intake when glucose was deficiency (Chen et al., 2016). Cancer cell lines, such as Panc-1, HPAF, Capan, HCT114 and HepG2, all could grow well at equivalent rates in fructose-containing media even though no glucose was available (Liu & Heaney, 2011). In human breast cancer cell line MDA-MB 468, fructose could accelerate cellular migration and invasion (Monzavi-Karbassi et al., 2010). In addition, MCF7 and MDA-MB-468 could take up more of fructose compared to the normal MCF10A cell line in glucose-deficiency medium (Gowrishankar et al., 2011). However, there is no convincing evidence showing that fructose could directly promote breast cancer development and progression. In this study, the *in vitro* and *in vivo* roles of fructose in breast cancers were investigated.

## Materials and Methods

### Cell culture

All cell lines were obtained from ATCC. MCF-7, MAD-MB-231, HeLa, HBL-100 and 3T3 cells were maintained in DMEM, and 4T1 and A549 cells were maintained in 1640, supplemented with 10% fetal bovine serum (Hyclone, USA) and 50 IU penicillin/streptomycin (Invitrogen, USA). MCF-10A cells were cultured in DMEM/F12 medium containing 10% horse serum, 20 ng/mL EGF, 0.5 mg/mL hydrocortisone, 100 ng/mL cholera toxin, 10 µg/mL insulin and 50IU penicillin/streptomycin. All cells were cultured inside an incubator containing 5% CO2 at 37 °C. In addition, glucose-free DMEM were obtained from Gibco, and fructose was obtained from Sigma. Considering minute quantity of glucose and fructose in media, the medium of glucose-free DMEM was glucose-free DMEM adding normal FCS in cell glucose-deficiency experiments, and substitutive nutrients, such as amino acids and fructose, were added to glucose-free DMEM.

### Plasmid construction

In this study, GLUT5 and KHK were down-regulated by shRNA, and pLKO.1-pure RNAi was used to construct shRNA. In order to obtain more accurate results, two efficient shRNA were used in this study. The shRNA sequences were as follows: shScr (Scramble shRNA): CCTAAGGTTAAGTCG CCCTCG; shKHK-1 (Human): CAGCGGATAGAGGAGCACAACTCGAGTT GTGCTCCTCTATCCGCTGC; shKHK-2 (Human): CATCATCAATGTGGTGG ACAACTCGAGTTGTCCACCACATTGATGATG; shmKHK-1 (Mouse): GCAGCGGATAGAGGAGCACAACTCGAGTTGTGCTCCTCTATCCGCTGC; shmKHK-2 (Mouse):CATCATCAATGTGGTGGACAACTCGAGTTGTCCACC ACATTGATGATG; shGLUT5-1 (Human): CCAATCGTTTGAGCTAATAACTC GAGTTATTAGCTCAAACGATTGGG; shGLUT5-2 (Human):TGTGAAGTGTT GTGTGTAACTCGAGTTACACACAACACTTCACAGC; shmGLUT5-1 (Mouse): CCTGCTGTTCAACAACATATTCTCGAGAATATGTTGTTGAACAGCAGG; shmGLUT5-2 (Mouse):CCCAATCGTTTGAGCTAATAACTCGAGTTATTAGC TCAAACGATTGGG.

### Lentivirus production

Viral packaging was done next. Briefly, plasmids shKHK and shGLUT5 were transfected into 293T cells through the calcium phosphate method. First, mixed calcium phosphate and plasmid into transfection medium on 293T cells for 5 h then replaced with fresh complete medium, and then medium was collected after transfection 48 h. Cancer cells were infected with the viruses, and then selected with puromycin.

### Western blot

After treatments as specified in the above, cells were washed twice with PBS and lysed in buffer (20 mM Tris-HCl, pH 7.5, 1 mM EDTA, 150 mM NaCl, 2.5 mM sodium pyrophosphate, 1% Triton X-100, 1 mM sodium vanadate, 1 mM b-glycer-ophosphate, 1 mM phenylmethyl-sulfonylfluoride and 1 mg/mL leupeptin). The same amounts of protein were loaded. The primary antibodies used in western blot were listed as follows: rabbit anti-KHK (Proteintech, Chicago, IL, USA), rabbit anti-GLUT5 (Abcam, Cambridge, MA, USA), mouse anti-*β*-actin (Proteintech). Western detection was using a Li-Cor Odyssey image reader. The anti-mouse immunoglobulin G (IgG) and anti-rabbit IgG secondary antibodies were from Li-Cor.

### Cell proliferation assay

For cell growth assay, we measured the cellular ATP value to determine cell proliferation using CellTiter-Glo^®^ Luminescent Cell Viability Assay kit (Promega, Fitchburg, WI, USA) according to the manual instructions. ATP was detected using a fluorophotometer. Cells were seeded into the 12-well plate, and 1 × 10^5^ in each well. After cell culturing 48 h, discard the culture medium, add normal cell culture medium for 2 h, so that the cell internal ATP content tends to stabilize, and then add 400 ul luciferase, shake for 5 min, pour the liquid into the EP tubes. The ATP was detected by the fluorescence detector.

### Cell colony formation assay

In the cloning assay, about 1.0 × 10^3^ cells were cultured in six plate wells. After treatments as specified in the results section, the cells were washed three times with PBS, poured into methanol at −20 °C for 6 min, and then washed three times with PBS, followed by fixation with crystal violet about 10 min.

### Wound-healing assay

About 5 × 10^5^ cells were added to each well of 6-well plate, and the next day cell monolayer was wounded by scratching with a 20 µL pipette tip. The cells were washed three times with PBS and added serum-free medium. The distances of cell migration were calculated by subtracting the distance between the wound edges at 24 h from the distances measured at 0 h.

### Animal experiment

We chose 4–5 weeks, 19–20 g, female BCLB/C mice, which were purchased from the Experimental Animal Center of Nanjing Medical. All animal experiments are followed to reduce the pain of animals. Mice were administered with a standard, housed and maintained in pathogen-free house in a 12:12 h light-dark cycle. Temperature and humidity were maintained at 24 ± 2 °C and 50 ± 5%, respectively. All mice were divided into three groups randomly: control group (water without fructose, *n* = 5), fructose-feeding group (15% fructose dissolved in water, *n* = 5), and glucose-feeding group (15% glucose dissolved in water, *n* = 5). Except for the different water, the standard laboratory chow was given to these three groups of mice. The same amount of 4T1/Luciferase cells was injected into the groin of all mice, and animal general status observation was monitored every day during the experiment. The second week of tumor formation, mice were imaged *in vivo*, focusing on the size and metastasis of primary tumor. The specific operation is, anesthetize the mouse, the subcutaneous injection of luciferase substrate, in 10 min after the start of photography. All animal studies were followed an approved protocol by Tianjin Cancer Institute and Hospital, in accordance with the principles and procedures outlined in the NIH Guide for the Care and Use of Laboratory Animals. The IACUC approval number is E2015093.

### Statistical analysis

SPSS 16.0 was used to evaluate the data, and the data were given as means ± SD. The standard two-tailed independent samples  *t*-test was performed to compare the differences of groups, and the significance level was defined as  *p* < 0.05 (* means *p* < 0.05, ** means *p* < 0.01). All experiments were repeated at least three times.

## Results

### Fructose rescues breast cancer cells from glucose deficiency-induced cell death

**Figure 1:**
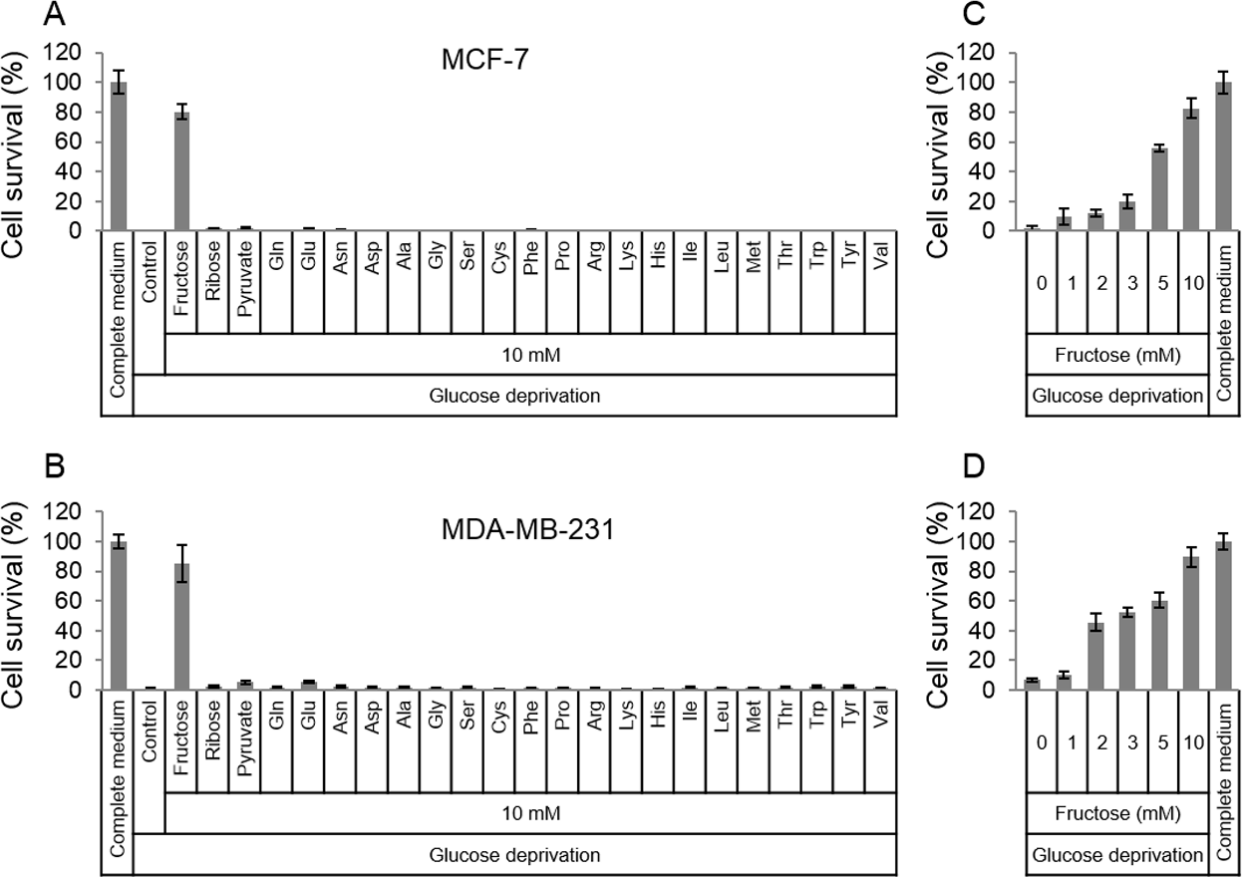
Fructose rescues cancer cells from glucose deficiency-induced cell death. (A) Cell survival of MCF-7 in common complete medium, no glucose medium, 10 mM fructose, ribose, pyruvate, Gln, Glu, Asn, sp, Ala, Gly, Ser, Cys, Phe, Pro, Arg, Lys, His, lle, Leu, Met, Thr, Trp, Tyr, or Val, respectively. (B) Cell survival of MDA-MB-231 in common complete medium, no glucose medium, 10 mM fructose, ribose, pyruvate, Gln, Glu, Asn, sp, Ala, Gly, Ser, Cys, Phe, Pro, Arg, Lys, His, lle, Leu, Met, Thr, Trp, Tyr, or Val, respectively. (C) Cell survival of MCF-7 in different fructose concentration (0, 1, 2, 3, 5, 10 mM) medium containing no glucose, or common complete medium. (D) Cell survival of MDA-MB-231 in different fructose concentration (0, 1, 2, 3, 5, 10 mM) medium containing no glucose, or common complete medium.

In order to survive in glucose-deficiency environment, cancer cells may use substitutive nutrients to support their proliferation. Given the above thought, common materials (fructose, ribose, pyruvate and amino acids) for cell survival were selected to detect if these nutrients in high dose could rescue cancer cells in glucose-deficiency medium. Two breast cancer cell lines, MCF-7 and MDA-MB-231, were cultured in the glucose-free medium that was supplemented with fructose, ribose, pyruvate or amino acids respectively. To our surprise, the results showed that fructose could robustly rescue both MCF-7 and MDA-MB-231 from cell death induced by glucose deficiency , compared to other nutrients (Figs. 1A and 1B). In addition, fructose could promote cell proliferation in a dose-dependent manner (0, 1, 2, 3, 5, 10 mM) in the condition of glucose deprivation. Furthermore, these cancer cells had similar growth rate in the glucose-free medium supplemented with 10 mM of fructose and in the complete medium containing 25 mM glucose (Figs. 1C and 1D). These data suggest that fructose is a potential energy source for cancer cells suffering from glucose insufficiency.

### Fructose does not support proliferation of non-tumor cells

**Figure 2:**
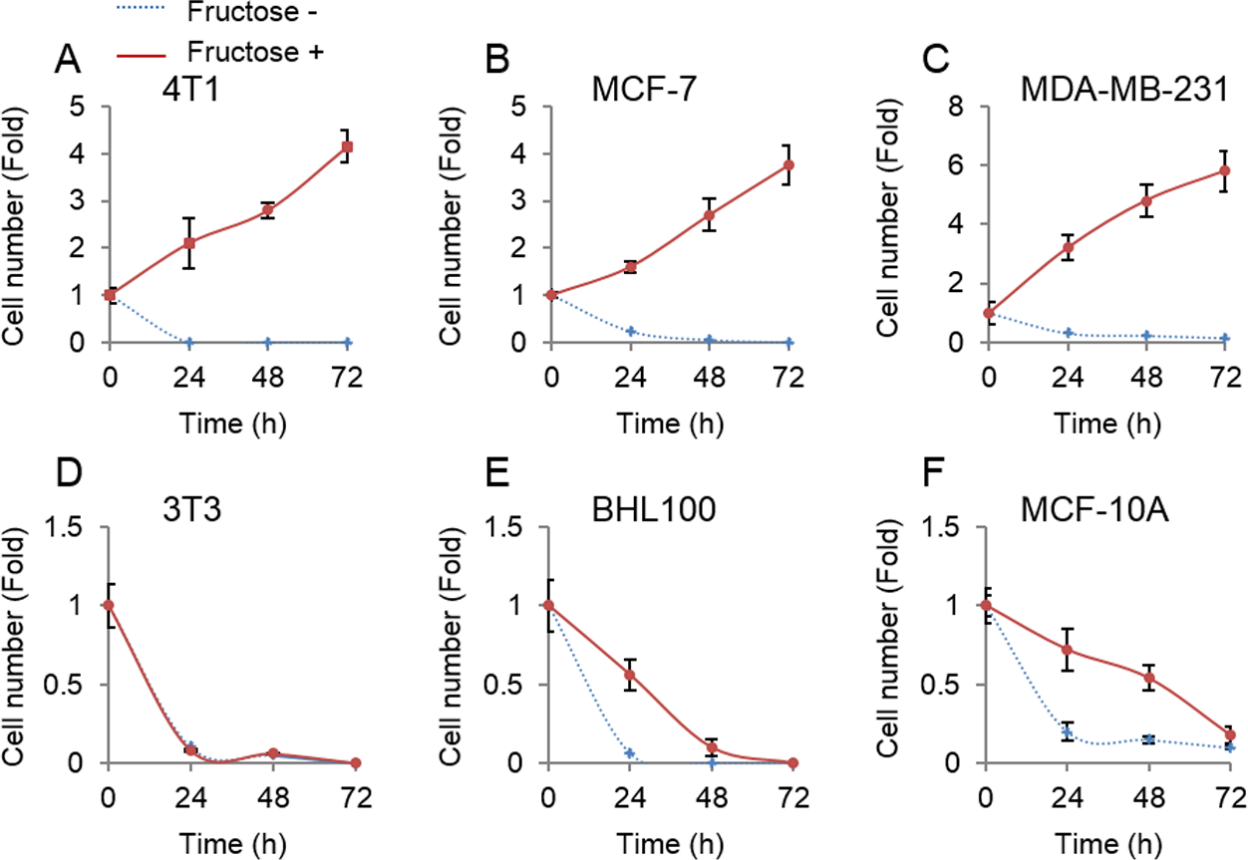
Tumor cells but not non-tumor cells use fructose. (A) Cell number of 4T1 in glucose-free medium supplemented with 10 mM of fructose (Fructose+) and no fructose (Fructose−) in 0, 24, 48, 72 h. (B) Cell number of MCF7 in Fructose+ medium and Fructose− medium in 0, 24, 48, 72 h. (C) Cell number of MAD-MB-231 in Fructose+ medium and Fructose− medium in 0, 24, 48, 72 h. (D) Cell number of 3T3 in Fructose+ medium and Fructose− medium in 0, 24, 48, 72 h. (E) Cell number of AT3 in Fructose+ medium and Fructose− medium in 0, 24, 48, 72 h. (F) Cell number HBL100 of in Fructose+ medium and Fructose− medium in 0, 24, 48, 72 h. (G) Cell number of MCF10A in Fructose+ medium and Fructose− medium in 0, 24, 48, 72 h.

Unlike glucose that was universally employed by all cells, fructose was mainly metabolized in the liver (Zhang et al., 2016). Therefore, we next tested whether non-tumor cells could proliferate upon supplement of fructose in the absence of glucose. Three non-tumor cell lines, mouse 3T3, human HBL100 and MCF-10A, and three breast cancer cell lines, mouse 4T1, and human MCF-7 and MDA-MB-231, were cultured in the glucose-free medium supplemented with 10 mM of fructose. Consistent with the forenamed results (Fig. 1), breast cancer cells proliferated well in the presence of fructose (Figs. 2A–2C). By contrast, our results showed that fructose did not support proliferation of non-tumor cells (Figs. 2D–2F). Therefore, breast cancer cells appear to obtain additional ability to utilize fructose, compared to their normal counterparts.

### Fructose and glucose exert similar effects on proliferation of breast cancer cells

**Figure 3:**
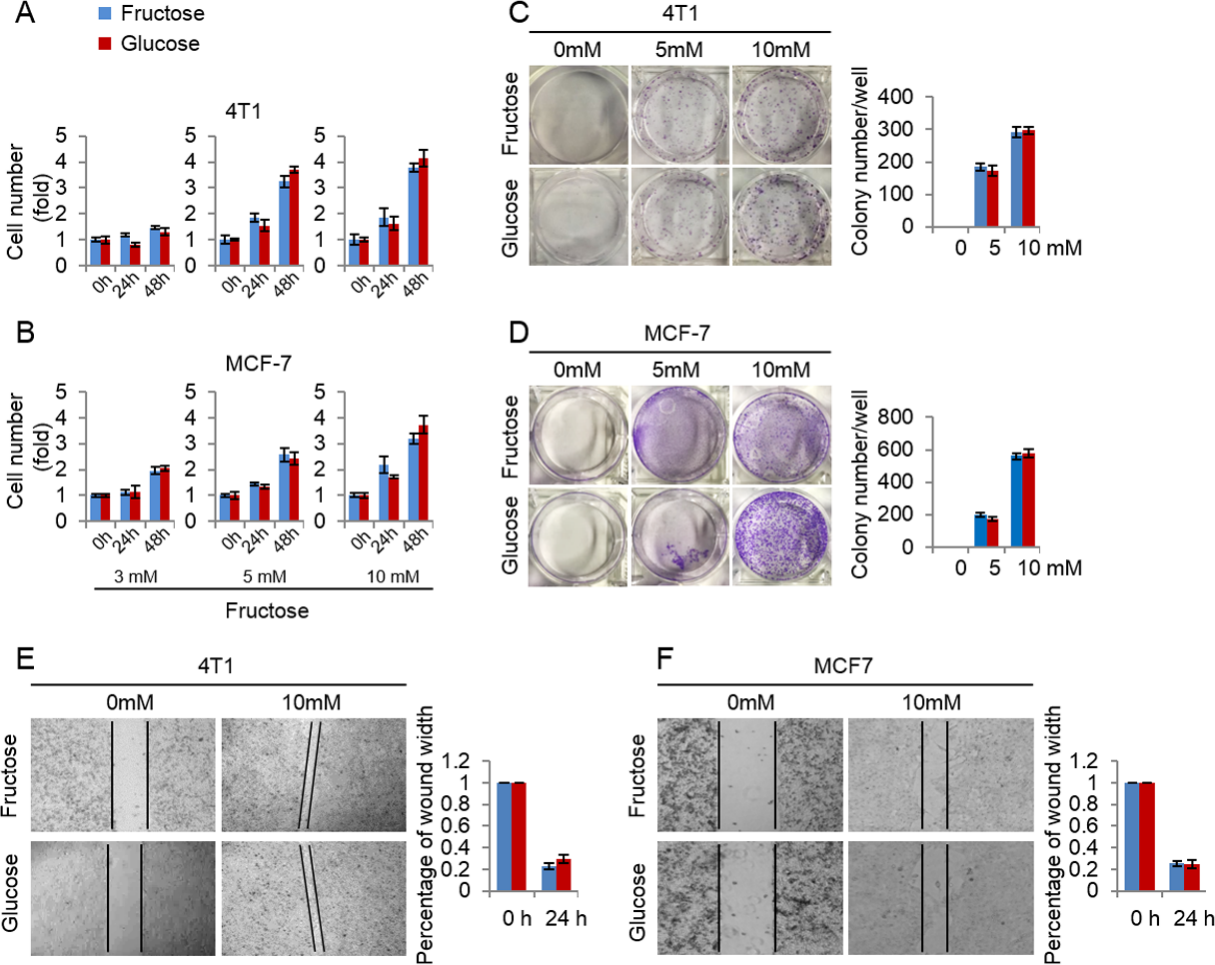
Fructose and glucose exert similar effects on proliferation and migration of cancer cells. (A) Cell number of 4T1 in the same concentration of fructose and glucose (3, 5, 10 mM) in 0, 24, 48 h. (B) Cell number of MCF-7 in the same concentration of fructose and glucose (3, 5, 10 mM) in 0, 24, 48 h. (C) Representative images of two-dimensional colony formation of 4T1 in the same concentration of fructose and glucose (0, 5, 10 mM). (D) Representative images of two-dimensional colony formation of MCF-7 in the same concentration of fructose and glucose (0, 5, 10 mM). (E) Wound healing test of 4T1 in the same concentration of fructose and glucose (0, 10 mM) in 24 h. (F) Wound healing test of MCF-7 in the same concentration of fructose and glucose (0, 10 mM) in 24 h.

Here we investigated the efficiency of fructose as a substitute for glucose in supporting breast cancer cell proliferation. First, we measured the growth rate of 4T1 and MCF-7 cells cultured in the media supplemented with the same concentrations (3, 5, 10 mM) of glucose or fructose respectively. As shown in Figs. 3A and 3B, glucose and fructose at each concentration displayed the similar effects on cell proliferation of 4T1 and MCF-7 at different times (24 h and 48 h). Next, the effects of fructose and glucose on the two-dimensional colony formation of 4T1 and MCF-7 cells were compared in the same condition. As shown in Figs. 3C and 3D, these cells showed the similar colony formation abilities in the medium supplemented with glucose or fructose. Finally, wound healing test was used to investigate whether fructose could promote the capability of migration, and our results showed that fructose supplement had a similar effect on cell migration for 4T1 and MCF-7 cells compared to glucose (Figs. 3E and 3F). Taken together, our data suggest that fructose is a functional substitute for glucose when glucose is insufficient.

### Fructose transport and subsequent metabolism is required for its role in supporting breast cancer cell proliferation

**Figure 4:**
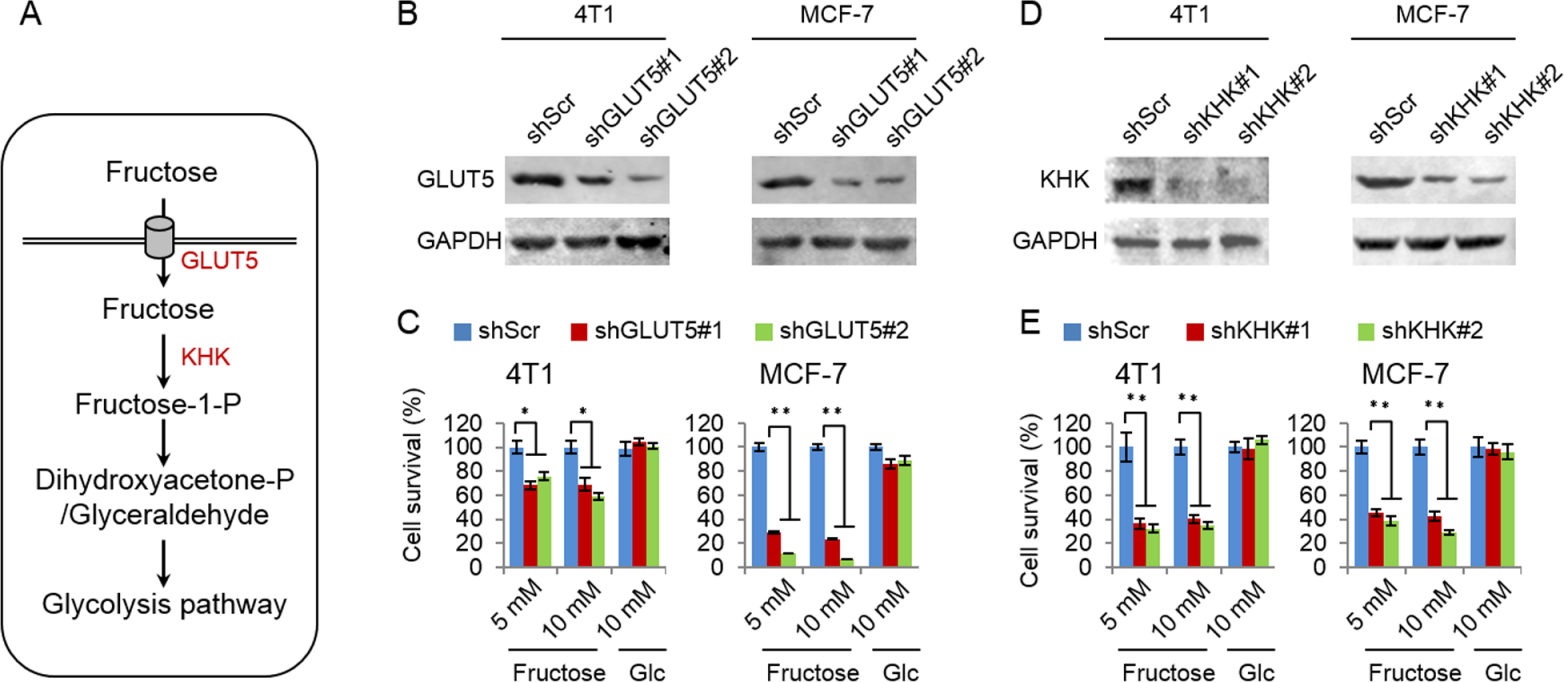
Fructose metabolism is required for its role in supporting cancer cell proliferation. (A) The possible metabolic pathway of fructose in cancer cells, GLUT5 is the main fructose transporter, and KHK is a key enzyme of fructose metabolism. (B) GLUT5 was down-regulated in 4T1 and MCF-7 using shRNA. (C) The effect of down-regulated GLUT5 on 4T1 and MCF-7 cell survival in fructose medium and glucose medium respectively. (D) KHK was down-regulated in 4T1 and MCF-7 using shRNA. (E) The effect of down-regulated KHK on 4T1 and MCF-7 cell survival in fructose medium and glucose medium respectively. (^∗^*p* < 0.05, ^∗∗^*p* < 0.01).

**Figure 5:**
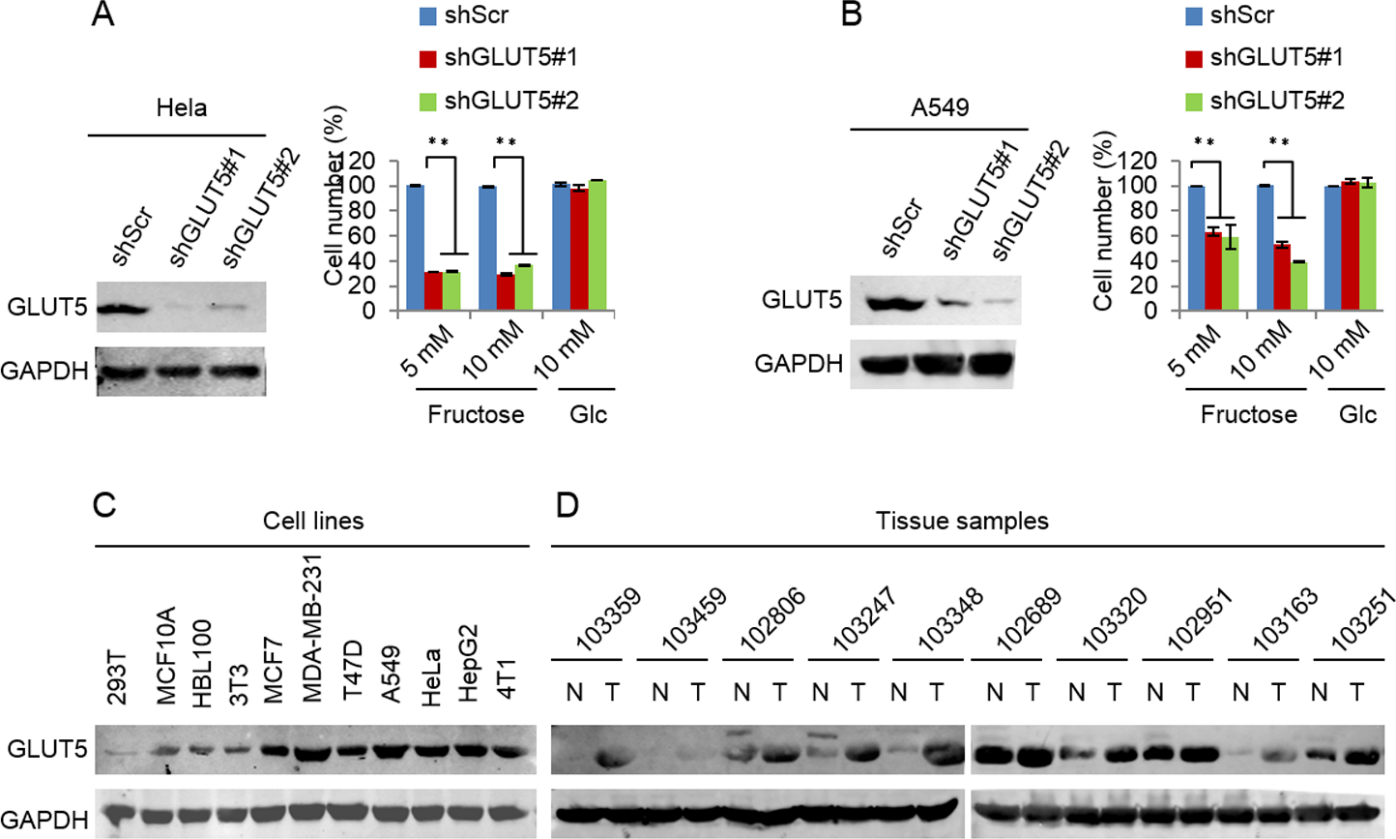
Down-regulated GLUT5 and its affection in other cancer cell lines. (A) GLUT5 was down-regulated in Hela using shRNA, and its affection on cell survival in fructose medium and glucose medium respectively. (B) GLUT5 was down-regulated in A549 using shRNA, and its effect on cell survival in fructose medium and glucose medium respectively. (C) Levels of GLUT5 expression in cell lines (293T, MCF10A, HBL100, 3T3, MCF7, MDA-MB-231, T47D, A549, Hela, HepG2, 4T1). (D) Levels of GLUT5 expression in breast cancer tissues (T) and their normal counterparts (N). (^∗^*p* < 0.05, ^∗∗^*p* < 0.01).

Glucose transport 5 (GLUT5) is mainly responsible for fructose absorption, and fructokinase (KHK) is a key enzyme involved in fructose catabolism (Fig. 4A) (Medina Villaamil et al., 2011; Patel et al., 2015b). To ascertain whether the intracellular catabolism of fructose was required for cancer cell proliferation, we determined the effects of GLUT5 and KHK on cell proliferation promoted by fructose using shRNA to knockdown GLUT5 or KHK. Our results showed that knockdown of KHK or GLUT5 significantly inhibited growth of 4T1 and MCF-7 cells in the fructose-containing medium but did not affect cell growth in the glucose-containing medium (Figs. 4B–4E). These results suggest that the intracellular catabolism is required for its role in supporting breast cancer cell proliferation. In addition, our results also demonstrated that fructose restored proliferation of HeLa and A549 cell lines depending on the presence of functional GLUT5 (Figs. 5A and 5B). Therefore, the effective utilization of fructose could be a ubiquitous feature of cancer cells. This speculation was further supported by our results that cancer cells including MCF-7, MDA-MB-231, T47D, A549, HeLa, HepG2 and 4T1 displayed obviously higher levels of GLUT5 than those non-tumor cells, such as 293T, MCF-10A, HBL100 and 3T3 (Fig. 5C). Furthermore, we measured the protein levels of GLUT5 in breast cancer tissues and normal counterparts of 10 patients. Similarly, GLUT5 was also hyper-expressed in tumor tissues compared to their normal counterparts (Fig. 5D). These observations underline the importance of fructose in the progress of cancers, including breast cancer.

### Fructose promotes metastasis of breast cancers

**Figure 6:**
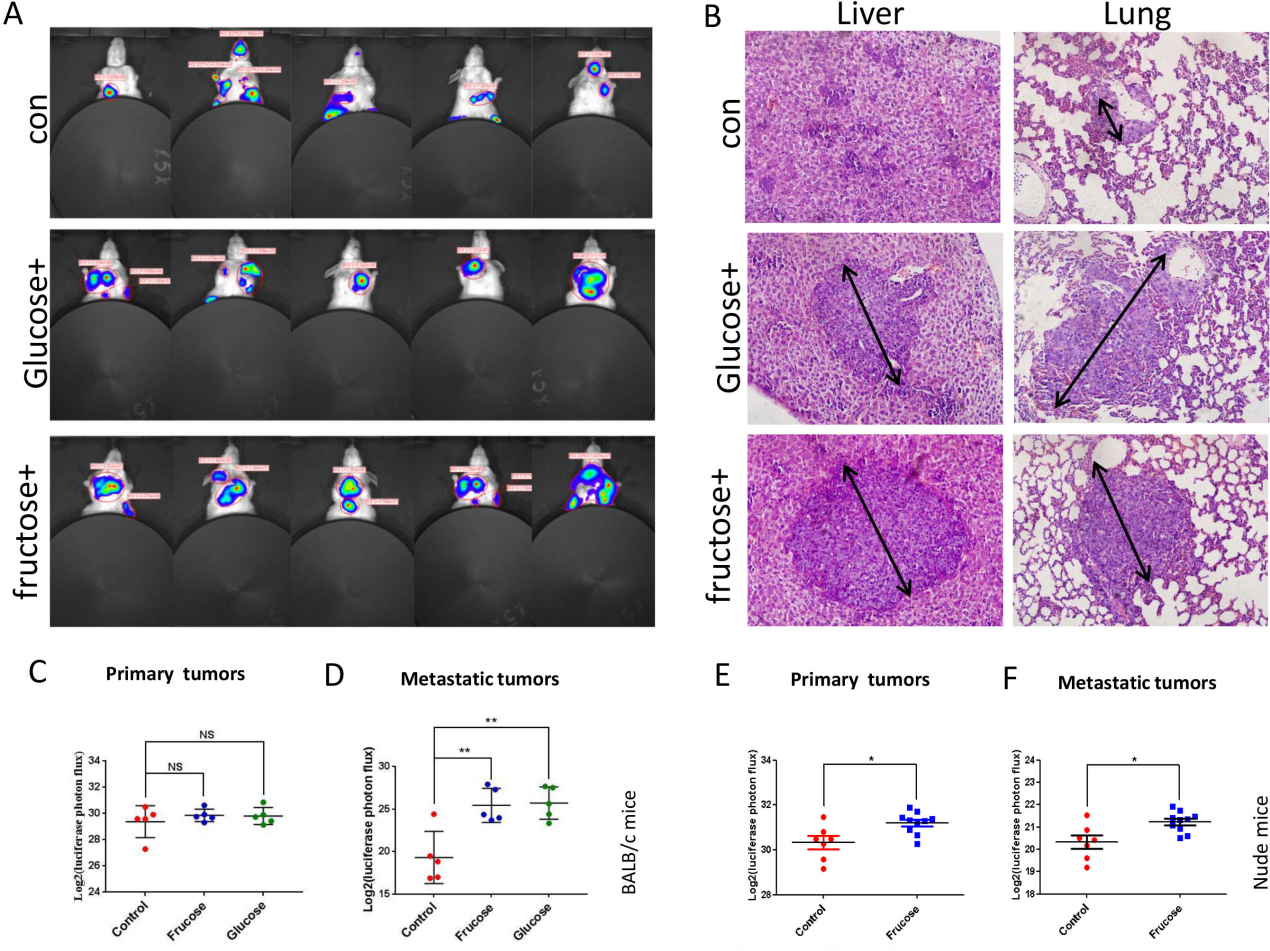
Fructose promotes metastasis of breast cancers *in vivo.* (A) Representative images of metastasis areas of high fructose diet group, glucose diet group and fructose or glucose-free group. (B) Hematoxylin and eosin stain in the liver and lungs. representative images were shown. (C) The different photon flux in primary tumor of high fructose diet BALB/c mice and control BALB/c mice and the different photon flux in primary tumor of glucose diet BALB/c mice and control BALB/c mice. (D) The different photon flux in metastatic tumor of high fructose diet BALB/c mice and control BALB/c mice. The same as the glucose and control group. (E) The different photon flux in primary tumor of high fructose diet nude mice and control nude mice. (F) The different photon flux in metastatic tumor of these two groups of nude mice.

Now, we sought to investigate the *in vivo* effect of fructose on breast cancer. BALB/C mice, subcutaneously injected with 4T1/Luciferase cells, were divided into three groups randomly: control group (water without fructose and glucose, *n* = 5), fructose-feeding group (15% fructose dissolved in water, *n* = 5), and glucose-feeding group (15% glucose dissolved in water, *n* = 5). All mice were fed the standard laboratory chow. The *in vivo* tumor growth and metastasis were supervised by luciferin photons every week. Our results showed that, although fructose diet and glucose diet did not affect the growth of primary of tumors, both diets significantly promoted the metastasis of 4T1 cells in BALB/c mice, and there was no significant difference between glucose diet and fructose diet in tumor growth and metastasis (Figs. 6A–6D). To further determine whether the immune system participated in this metastasis process, nude mice subcutaneously injected with 4T1/Luciferase cells were used. Fructose also accelerated the metastasis of 4T1 cells in nude mice, and moreover it significantly increased primary tumor growth (Figs. 6E and 6F). These data suggested that fructose played a considerable role in promoting the malignance of cancers.

## Discussion

Fructose is one of the most common blood sugars second to glucose, and it widely exists in our foods, such as corns, fruits and honey. In addition, the markedly increasing dietary fructose are used as a common sweetener in the food production (Li et al., 2016). Recently, fructose is tightly linked to many metabolic diseases, such as non-alcoholic fatty liver disease, insulin resistance, diabetes, hypertension, hyperlipidemia, cardiovascular and cerebrovascular diseases, obesity and chronic consumption (Reusch, 2002; Sacks, 2004; Spinler, 2006), but the clear relationship between fructose and tumor has remained to be explored. In this study, we revealed that fructose was functionally used by breast cancer cells but not by non-tumor cells. Meanwhile, fructose was found to support *in vitro* proliferation, colony formation and migration of cancer cells as effective as glucose, and promote *in vivo* metastasis of breast cancers. We believe that this study would arise more attention about the relationship of cancer development and fructose consumption.

Due to the increased requirement of glucose consumption, proliferating cancer cells often suffer from glucose insufficiency (Dai et al., 2016; Darnell Jr, 2010). Therefore, how to keep proliferating in the condition of glucose deficiency is a big challenge faced by *in vivo* cancer cells. To utilize some functional substitutes for glucose should be a feasible choice for cancer cells. Indeed, tumor cells could use many kinds of nutrients, such as glutamine and free fatty acid, to rescue cells from death induced by glucose deficiency (Kim et al., 2017). In this study, several materials (Fructose, Ribose, Pyruvate, and every animo acid) for cell survival were selected to detect which material was more efficient for cell proliferation in glucose deficiency, and the results showed that fructose performed the most powerful efficiency in cell survival and proliferation. Meanwhile, the same concentration of fructose and glucose got the similar effect in cell biological behavior, including cell proliferation, colony formation and cell migration. Furthermore, animal experiments of this study showed that fructose could accelerate the growth and metastasis of breast cancer cells in both nude mice and BCLB/C mice, which also suggested that the role of fructose in promoting cancer progression was mainly by the metabolic pathways but not the immune system, and the specific mechanism of these need more studies to explore. In fact, there were some investigations showing that high fructose intake was associated with an increase in pancreatic cancer risk and promoted the deterioration of pancreatic cancer (Liu et al., 2010). Although previous study has reported that increased dietary fructose uptake was able to enhance breast tumor growth and promote metastasis (Jiang et al., 2016), our study further confirmed the role of fructose in breast tumor growth and metastasis *in vivo* using whole-body optical imaging system and revealed the potential mechanism.

Since fructose is not competitively used by the normal cells, it could be relatively specific to and selectively used by cancer cells *in vivo*. Correspondingly, a fructose transporter, GLUT5, was found to over-express in cancer cells and breast cancer tissues, consistent with a previous report (Chen et al., 2016). Moreover, convincing evidence demonstrated that high-fructose diet induced high expression of GLUT5 and KHK expression, which in turn enhanced the efficiency of fructose absorption (Patel et al., 2015a). Our data showed that GLUT5 knockdown blocked fructose utilization by cancer cells, and thus GLUT5 inhibitors could be a potential anti-cancerous drug that suppresses the metastasis of cancers. However, such drugs might not deserve development because cancer patients can easily have a low-fructose diet by avoiding eating corns, some sweet fruits, honey and other food containing high level of fructose. In view of the potential stimulation of fructose to the metastasis of cancers, the clinic sodium fructose diphosphate for injection may not be used in tumor patients.

Our findings review the relationship between increased dietary fructose and breast cancer risk, and provide important insights into recent epidemiological studies and present preliminary insights into the potential therapeutic strategies that may eliminate fructose-mediated breast cancer. Further investigation of the relationship between fructose consumption and cancer risk is critical, because dietary intake is highly modifiable, and it can represent a primary opportunity to prevent cancer and be used as diagnostic criteria for cancer prognosis.

## Conclusions

In conclusion, our data show that fructose can be specifically used by breast cancer cells as the substitute for glucose, and the high-fructose diet could accelerate the progress of breast cancer *in vivo*.

##  Supplemental Information

10.7717/peerj.3804/supp-1 Supplemental Information 1 Measuring the ATP values for each cell are above Supplements the data for Fig. 1A by measuring the value of ATP response cells. The ATP values for each cell are above.

10.7717/peerj.3804/supp-2 Supplemental Information 2 Measuring the value of ATP of each cells Supplements the data for Fig. 1B by measuring the value of ATP response cells. The ATP values for each cell are above.

10.7717/peerj.3804/supp-3 Supplemental Information 3 Measuring the value of ATP of each cells Supplements the data for Figs. 2A–2C by measuring the value of ATP response cells. The ATP values for each cell are above.

10.7717/peerj.3804/supp-4 Supplemental Information 4 Measuring the value of ATP of each cells Supplements the data for Figs. 2D–2F by measuring the value of ATP response cells. The ATP values for each cell are above.

10.7717/peerj.3804/supp-5 Supplemental Information 5 Measuring the value of ATP of each cells Supplements the data for Figs. 3A and 3B by measuring the value of ATP response cells. The ATP values for each cell are above.

10.7717/peerj.3804/supp-6 Supplemental Information 6 Measuring the value of ATP of each cells Supplements the data for Fig. 4C by measuring the value of ATP response cells. The ATP values for each cell are above.

10.7717/peerj.3804/supp-7 Supplemental Information 7 Measuring the value of ATP of each cells Supplements the data for Fig. 4E by measuring the value of ATP response cells. The ATP values for each cell are above.

10.7717/peerj.3804/supp-8 Supplemental Information 8 Measuring the value of ATP of each cells Supplements the data for Figs. 5A and 5B by measuring the value of ATP response cells. The ATP values for each cell are above.

10.7717/peerj.3804/supp-9 Supplemental Information 9 The WB of Fig. 4 Odyssey Infrared Fluorescence Imaging System was used to text the WB. The secondary antibody is the fluorescent antibody.

10.7717/peerj.3804/supp-10 Supplemental Information 10 The WB of Fig. 5

10.7717/peerj.3804/supp-11 Supplemental Information 11 The volume of the primary tumor The volume of the primary tumor of control BALB/c mice, high fructose diet and glucose diet BALB/c mice.

10.7717/peerj.3804/supp-12 Supplemental Information 12 The volume of the primary tumor The volume of the primary tumor of control nude mice and high fructose diet nude mice.

## Additional Information and Declarations

### Data Availability

The following information was supplied regarding data availability: The raw data has been included as Supplementary Figures.
