# Supplementary figures and images for: Increased utilization of fructose has a positive effect on the development of breast cancer
Source: PeerJ. 2017 Sep 27;5:e3804. doi: 10.7717/peerj.3804 (PMC5622605; doi:10.7717/peerj.3804)

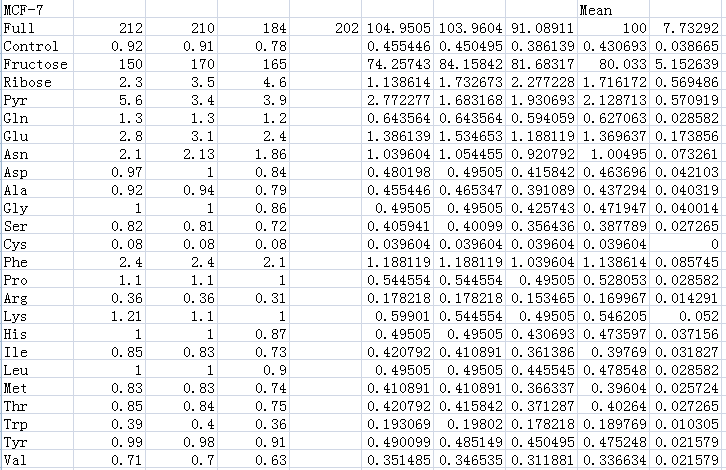

Supplement: Supplemental Information 1 — Supplements the data for Fig. 1A by measuring the value of ATP response cells. The ATP values for each cell are above. [file peerj-05-3804-s001.png]

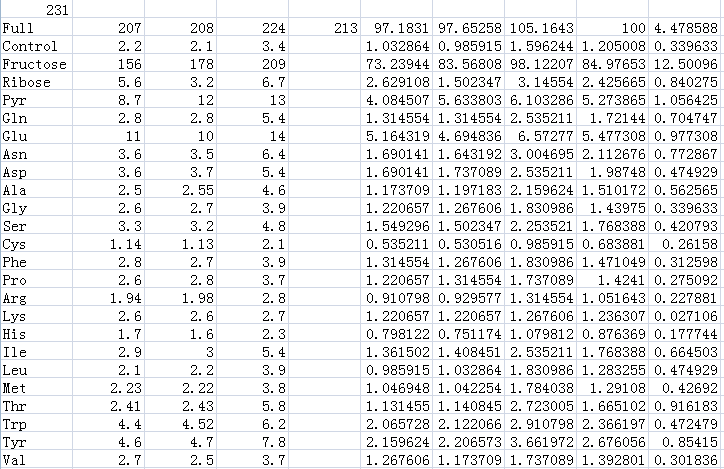

Supplement: Supplemental Information 2 — Supplements the data for Fig. 1B by measuring the value of ATP response cells. The ATP values for each cell are above. [file peerj-05-3804-s002.png]

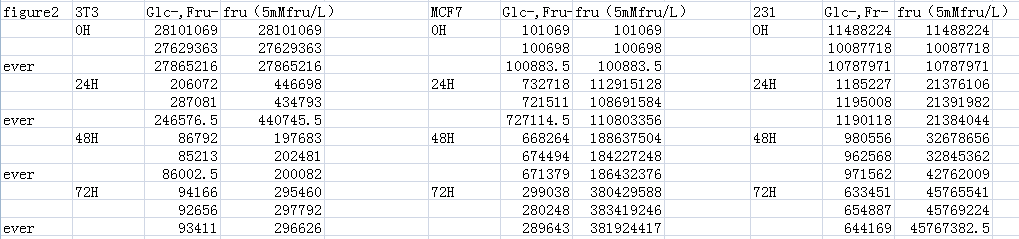

Supplement: Supplemental Information 3 — Supplements the data for Figs. 2A–2C by measuring the value of ATP response cells. The ATP values for each cell are above. [file peerj-05-3804-s003.png]

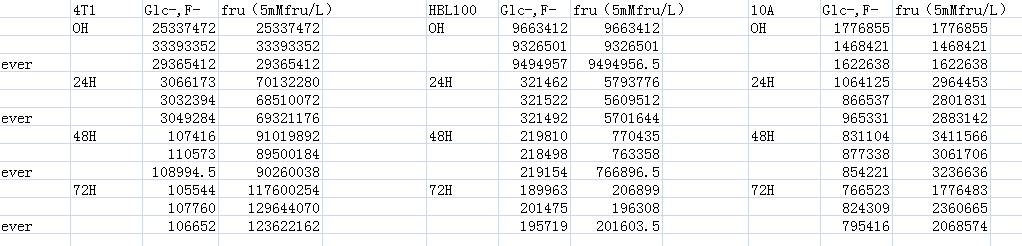

Supplement: Supplemental Information 4 — Supplements the data for Figs. 2D–2F by measuring the value of ATP response cells. The ATP values for each cell are above. [file peerj-05-3804-s004.png]

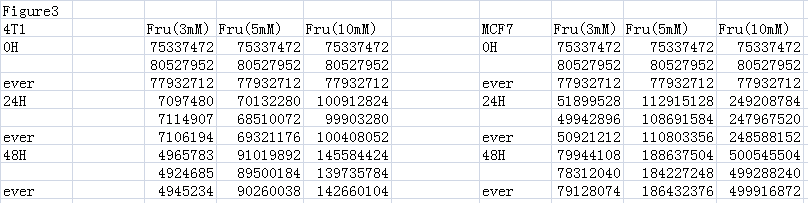

Supplement: Supplemental Information 5 — Supplements the data for Figs. 3A and 3B by measuring the value of ATP response cells. The ATP values for each cell are above. [file peerj-05-3804-s005.png]

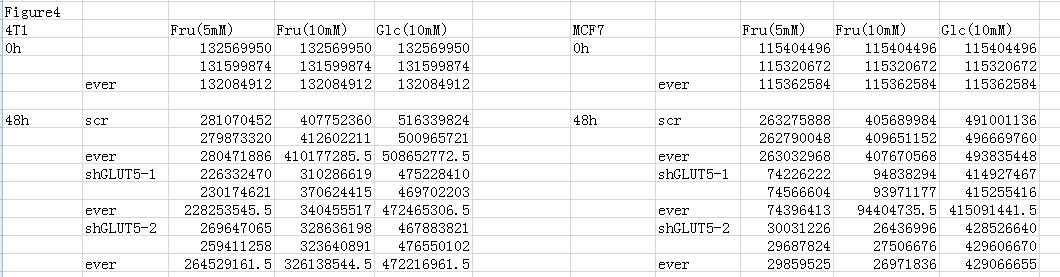

Supplement: Supplemental Information 6 — Supplements the data for Fig. 4C by measuring the value of ATP response cells. The ATP values for each cell are above. [file peerj-05-3804-s006.png]

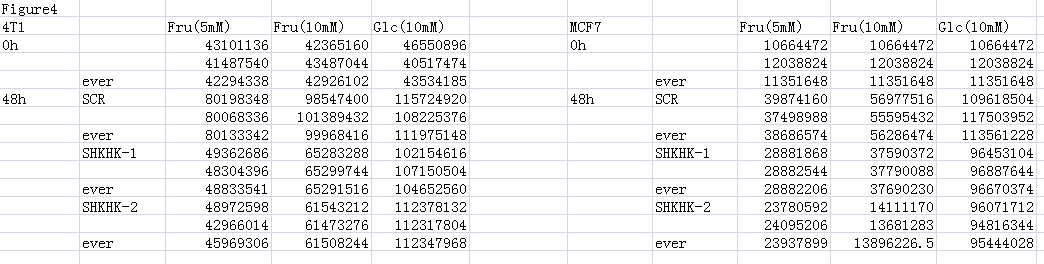

Supplement: Supplemental Information 7 — Supplements the data for Fig. 4E by measuring the value of ATP response cells. The ATP values for each cell are above. [file peerj-05-3804-s007.png]

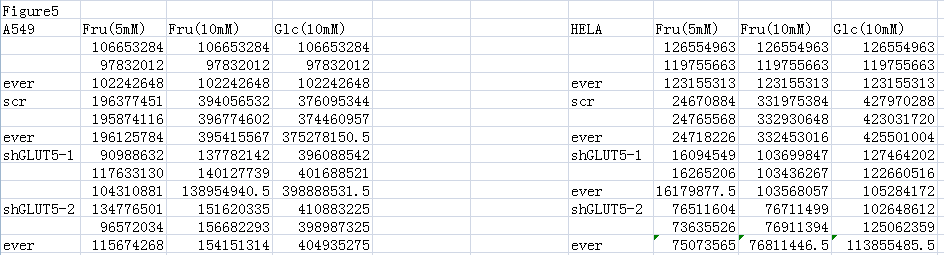

Supplement: Supplemental Information 8 — Supplements the data for Figs. 5A and 5B by measuring the value of ATP response cells. The ATP values for each cell are above. [file peerj-05-3804-s008.png]

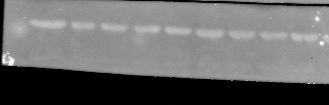

Supplement: Supplemental Information 9 — Odyssey Infrared Fluorescence Imaging System was used to text the WB. The secondary antibody is the fluorescent antibody. [file peerj-05-3804-s009.zip › figure4 wb/shGLUT5 in different cell lines 4T1 MCF7/800 GAPDH.TIF]

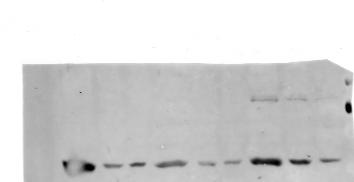

Supplement: Supplemental Information 9 — Odyssey Infrared Fluorescence Imaging System was used to text the WB. The secondary antibody is the fluorescent antibody. [file peerj-05-3804-s009.zip › figure4 wb/shGLUT5 in different cell lines 4T1 MCF7/800 shGLUT5 in 4T1 and MCF7.TIF]

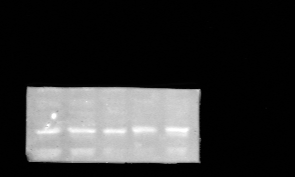

Supplement: Supplemental Information 9 — Odyssey Infrared Fluorescence Imaging System was used to text the WB. The secondary antibody is the fluorescent antibody. [file peerj-05-3804-s009.zip › figure4 wb/shKHK in different cell lines/800 4T1.TIF]

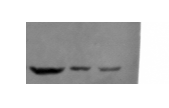

Supplement: Supplemental Information 9 — Odyssey Infrared Fluorescence Imaging System was used to text the WB. The secondary antibody is the fluorescent antibody. [file peerj-05-3804-s009.zip › figure4 wb/shKHK in different cell lines/800 MCF7 KHK.tif]

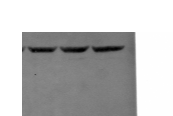

Supplement: Supplemental Information 9 — Odyssey Infrared Fluorescence Imaging System was used to text the WB. The secondary antibody is the fluorescent antibody. [file peerj-05-3804-s009.zip › figure4 wb/shKHK in different cell lines/800 MCF7 GAPDH.tif]

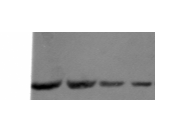

Supplement: Supplemental Information 10 [file peerj-05-3804-s010.zip › figure5 wb/shGLUT5 in Hela and a549/700 HELA GLUT5.tif]

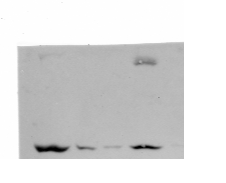

Supplement: Supplemental Information 10 [file peerj-05-3804-s010.zip › figure5 wb/shGLUT5 in Hela and a549/800 a549shGLUT5.tif]

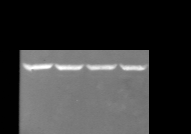

Supplement: Supplemental Information 10 [file peerj-05-3804-s010.zip › figure5 wb/shGLUT5 in Hela and a549/800 HELA GAPDH.tif]

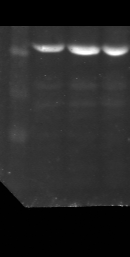

Supplement: Supplemental Information 10 [file peerj-05-3804-s010.zip › figure5 wb/shGLUT5 in Hela and a549/800 GAPDH A549.tif]

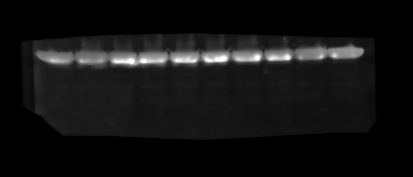

Supplement: Supplemental Information 10 [file peerj-05-3804-s010.zip › figure5 wb/the levels of GLUT5expression in breast cancer tissues/800 GAPDH(2).TIF]

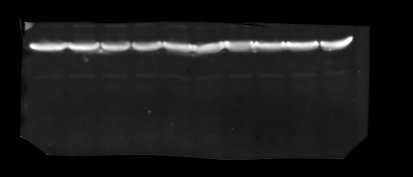

Supplement: Supplemental Information 10 [file peerj-05-3804-s010.zip › figure5 wb/the levels of GLUT5expression in breast cancer tissues/800 GAPPDH.TIF]

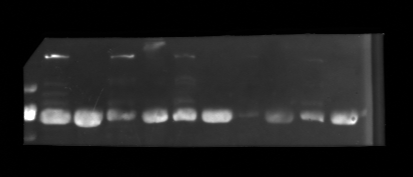

Supplement: Supplemental Information 10 [file peerj-05-3804-s010.zip › figure5 wb/the levels of GLUT5expression in breast cancer tissues/800 (2).TIF]

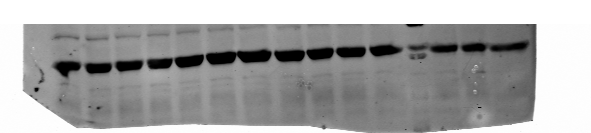

Supplement: Supplemental Information 10 [file peerj-05-3804-s010.zip › figure5 wb/the levels of GLUT5expression in cell lines/800 GAPDH.TIF]

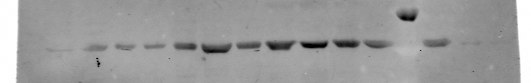

Supplement: Supplemental Information 10 [file peerj-05-3804-s010.zip › figure5 wb/the levels of GLUT5expression in cell lines/800 GLUT5.TIF]
